# Interactions by Disorder – A Matter of Context

**DOI:** 10.3389/fmolb.2020.00110

**Published:** 2020-06-16

**Authors:** Katrine Bugge, Inna Brakti, Catarina B. Fernandes, Jesper E. Dreier, Jeppe E. Lundsgaard, Johan G. Olsen, Karen Skriver, Birthe B. Kragelund

**Affiliations:** ^1^REPIN, Department of Biology, University of Copenhagen, Copenhagen, Denmark; ^2^Structural Biology and NMR Laboratory, Department of Biology, University of Copenhagen, Copenhagen, Denmark

**Keywords:** IDP, SLiM, protein interactions, context, flanking region, intrinsically disordered proteins, ensemble redistribution, interaction mechanism

## Abstract

Living organisms depend on timely and organized interactions between proteins linked in interactomes of high complexity. The recent increased precision by which protein interactions can be studied, and the enclosure of intrinsic structural disorder, suggest that it is time to zoom out and embrace protein interactions beyond the most central points of physical encounter. The present paper discusses protein–protein interactions in the view of structural disorder with an emphasis on flanking regions and contexts of disorder-based interactions. Context constitutes an overarching concept being of physicochemical, biomolecular, and physiological nature, but it also includes the immediate molecular context of the interaction. For intrinsically disordered proteins, which often function by exploiting short linear motifs, context contributes in highly regulatory and decisive manners and constitute a yet largely unrecognized source of interaction potential in a multitude of biological processes. Through selected examples, this review emphasizes how multivalency, charges and charge clusters, hydrophobic patches, dynamics, energetic frustration, and ensemble redistribution of flanking regions or disordered contexts are emerging as important contributors to allosteric regulation, positive and negative cooperativity, feedback regulation and negative selection in binding. The review emphasizes that understanding context, and in particular the role the molecular disordered context and flanking regions take on in protein interactions, constitute an untapped well of energetic modulation potential, also of relevance to drug discovery and development.

## Introduction

Living organisms depend on self-orchestrated interactions between molecules linked in interactomes of enormous complexity (Mosca et al., 2013; Cafarelli et al., 2017). In these, protein–protein interactions must happen with a precision and in a timely manner that secure specificity and fidelity of the interactome. Protein interactions depend on electrostatics, hydrophobicity, dynamics and complementarity, as well as regulatory mechanisms enabling the complexes to trigger and assert the functions required, be it catalysis, signal transduction, transcription, mechanical structure or something else. There may even be a need for these aspects to function under different physicochemical and physiological circumstances. Importantly, proteins with intrinsically disordered properties, whether it is a completely intrinsically disordered protein (IDP) or a protein with an intrinsically disordered region (IDR), are key to network fidelity (Dosztányi et al., 2006; Oldfield et al., 2008; Staby et al., 2017; Uversky, 2018), and they exist in ensembles of almost isoenergetic states. Their complexity cannot be described by a single type of experiment but requires several complementary observations. Over the years, the range of amenable observations has broadened, the precision of the measurements improved, and the theoretical understanding of what an IDP is and how macromolecules interact is increasing rapidly (Kragelund and Skriver, 2020). It is therefore time to zoom out and embrace protein complex formation and function beyond the most central points of physical encounter and take the environment and context into account in a much broader term.

### Context Is Multidimensional

The context of IDP interactions includes time and space, as well as the disordered chain in which the contact points are embedded. Traditionally, we have mostly considered context in relation to three-dimensional structures, but for IDPs this fails to provide us with insight into how context influences binding. Compared to structured proteins, IDP ensembles are more sensitive to changes in their settings, and by extension, the same is true for the interactions they engage in.

For many IDPs, their interactions – and hence their contacts to their partner molecule – are made via short, sequence-embedded motifs of limited information (Sharma et al., 2014; Davey et al., 2015). Short linear motifs (SLiMs) are more prevalent in IDPs, and the human proteome is estimated to contain > 100.000 – possibly a million – SLiM instances (Davey et al., 2012; Tompa et al., 2014). SLiMs are typically 6–12 residues long and can usually be recognized by patterns of conserved residues within an otherwise sparsely conserved sequence stretch (Davey et al., 2012; Sharma et al., 2014; Krystkowiak and Davey, 2017), although sometimes, they are so degenerate as to go unnoticed by sequence analysis alone. The SLiM is the central anchoring site for many IDP interactions and a recent review discusses the molecular details of SLiM-based affinity and specificity (Ivarsson and Jemth, 2019).

**FIGURE 1:**
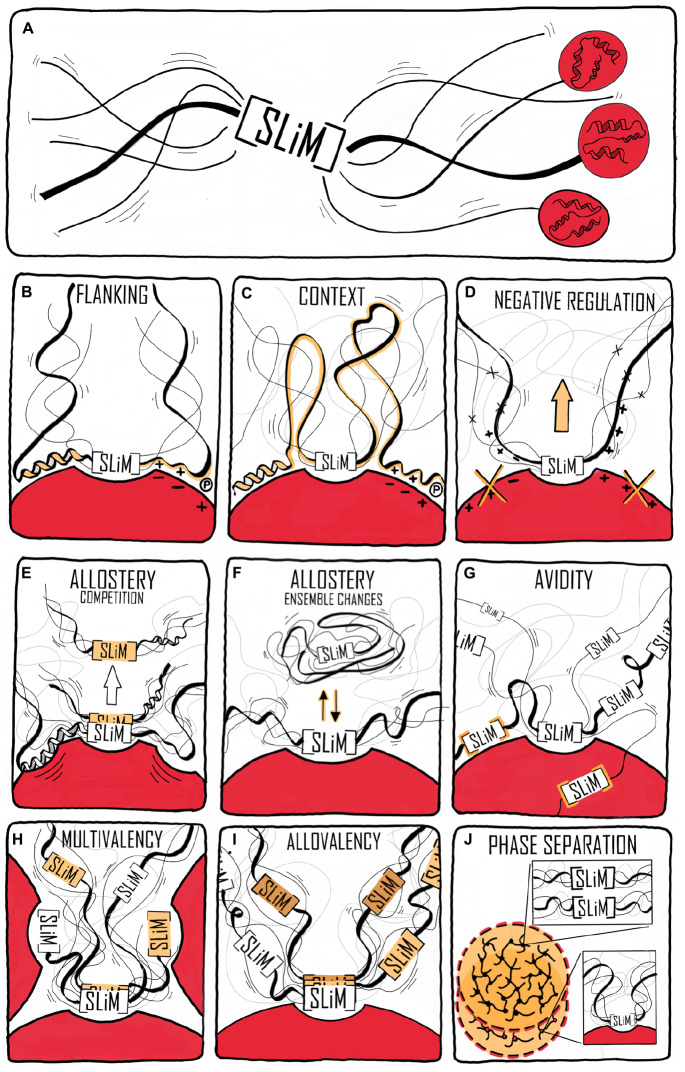
Context contribution to disorder-based protein interactions. **(A)** A binding region within an IDP illustrated by a single SLiM. The binding region is embedded in a disordered chain in which additional features are present that can affect the interaction. The red spheres with helical structures illustrate that the IDR may be part of a modular protein and connected to folded domains. **(B)** Flanking regions can modulate IDP binding by, e.g., charge complementarity, secondary structure formation, and phosphorylation (illustrated by an encircled P). **(C)** Binding regions distant to the central binding site can contextually modulate binding. **(D)** Negative selection by means of mismatching flanking region properties. **(E)** Allosteric regulation through the flanking region via conformational changes in the folded partner (red) induced by IDP binding. This may also lead to partner selection as indicated by the second SLiM (in orange) leaving the binding site. **(F)** Allosteric regulation by ensemble redistribution of the disordered chain, here illustrated by changes in the degree of compaction. **(G)** Avidity by additional SLiMs or binding sites within the disordered chain binding outside the (or one of the) central contact point(s) (orange). **(H)** Multivalency by additional SLiMs or binding sites binding to different proteins resulting in dynamic binding illustrated by three chains with different colored SLiMs. **(I)** Contextual allovalency. Several SLiMs within the same disordered chain bind to the same binding site on the target protein – one at the time – and increase affinity through allovalency effects, here illustrated by three different chains of the ensemble, **(J)** Liquid-liquid phase separation (LLPS) by multiple SLiMs (top droplet and top zoom) or by SLiM:domain interactions (bottom droplet and bottom zoom). The red spheres represent a folded binding partner in all figures.

In recent years, SLiMs have been studied extensively applying different methods, among them especially bioinformatics (Krystkowiak and Davey, 2017), but also by several different other structural and biochemical methods (Kragelund and Skriver, 2020). Through these studies, it has become clear that the properties of a complex (that is affinity, specificity, structure, kinetics, and thermodynamics) cannot always be explained solely by focusing on the SLiM interaction, but that the entire context of disorder and the presence of folded domains need to be considered (Figure 1A). For example, affinities have been modulated by changing the structural context outside of a SLiM via single residue mutations promoting secondary structure elements present in the bound state (Iešmantavičius et al., 2014) or by charge properties next to the SLiM (Stein and Aloy, 2008; Hertz et al., 2016; Palopoli et al., 2018; Prestel et al., 2019). Conformational heterogeneity, characteristic of many ID-based interactions (Fuxreiter, 2019), is also regulated by residues outside the SLiM-binding site, where they form transient heterogeneous contacts, which may facilitate partner-templated coupled folding and binding (Toto et al., 2016). No formal terminology exists for describing the context, and the immediate N- and C-terminal context of the SLiM will therefore here be referred to as the SLiM flanking regions. The boundary between what we define as SLiM and flanking regions is a continuum, likewise, so is the boundary between flanking regions and the remainder of the chain context. Limiting the flanking regions to, say, 20 residues on either side probably makes sense for most proteins, but some SLiMs may have shorter SLiM-like flanking regions of relevance (Figure 1B). In other cases, properties belonging to the chain may also contribute to binding affinity and specificity, and not necessarily through direct contact to the binding partner (Figure 1C). We will consider these as part of the context and not part of the flanking regions. Clearly, flanking regions are also part of the context and the boundary between flanks and the greater context is likely system dependent.

The present paper discusses disordered protein–protein interactions with a focus on context, and with a special emphasis on the disordered chain and how its properties affect protein interactions. Context has a broad meaning, being the physicochemical environment (pH, viscosity, pressure, salt concentration, etc.), the biomolecular environment (interactors and location), or the physiological environment (cell-type, cell cycle, stress, etc.), but it is also the *intra*molecular context of the interaction; that is the peptide chain to which the interaction site belongs. Due to their abundance, SLiMs serve as important models/platform for the understanding of context in relation to disorder. However, interactions between IDPs may not necessarily be SLiM-based, but can also be mediated by disorder itself. This review highlights how flanking regions and the disordered contexts are emerging as important contributors to allosteric regulation, positive and negative cooperativity, feed-back regulation and negative selection in disorder-based binding and it poses important outstanding questions that need attention to enable a full comprehension of how disorder-based interactions operate.

## The Context of Time and Space

Before we engage in discussing the chain properties of disorder-based interactions, it is important to recognize that IDPs function in different compartments of the cell, are distributed in different tissue types and can be both extracellular and intracellular. They operate in timed manners covering many different time scales including evolution, development, aging and temporal regulation needed for maintaining homeostasis and turnover during cell cycles. Such settings provide a spatiotemporal context capable of modulating IDP interactions.

### Isoforms Allow Context Adaptation

The context of development may affect IDP interactions through, e.g., the specific expression of isoforms at different developmental stages that differ in their ability to interact with partners, as shown for the Ubx transcription factors (TFs), which are key players in *Drosophila* embryonic development (López et al., 1996; Hsiao et al., 2014). Similarly, many isoforms that differ in the length and sequence context of their IDRs are distributed between different cell types and organelles, and experience different operating contexts. The sodium-proton exchanger (NHE) family is an example of this. NHEs are membrane proteins, and the nine isoforms have similar membrane transport functions, but have IDRs of different lengths with varying SLiM content and sequence (Nørholm et al., 2011; Hendus-Altenburger et al., 2014), suggesting that the mechanisms and regulations underlying their function in the membrane, differ. Some isoforms are brain specific, e.g., NHE6 and NHE9 (Schwede et al., 2014; Zhao et al., 2016), and some are ubiquitously located in the plasma membrane, such as NHE1, while other isoforms are localized in specific organellar membranes (Ohgaki et al., 2011; Prasad and Rao, 2015; Pedersen and Counillon, 2019). Thus, despite similar functions, the spatial context in which these proteins carry out their function is different, as a result of their different IDRs.

### Context Fluctuates With Time

**FIGURE 2:**
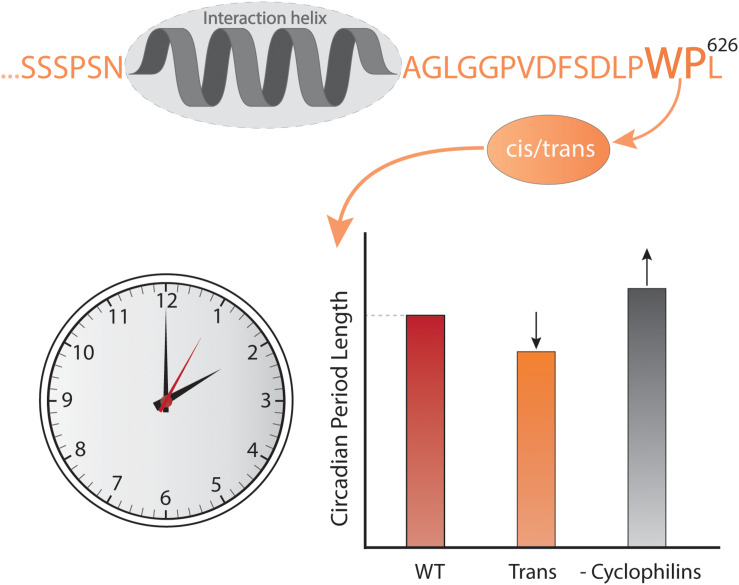
Context and time. The C-terminal conformational switch, flanking a central helical region involved in interactions with CBP/p300 and CRY1 in the disordered transcriptional activation domain of BMAL1, involves *cis*/*trans* isomerization around a Trp-Pro imide bond, which modulates circadian timing. Locking the switch into the *trans* conformation results in a shorter circadian period compared to the wild-type, while inhibiting cyclophilins, which accelerate the interconversion between isomers, lengthens the circadian period in a dose-dependent manner (Gustafson et al., 2017).

Time also affects the context in which IDPs work. At the long time scales, evolution changes the context and although sequences develop, emerging evidence suggests that order-disorder patterns are evolutionary conserved whereas sequence is not, as exemplified by the plant NAM, ATAF, and CUC (NAC) TFs (Christensen et al., 2019). A comparison of five Ubx TF orthologs spanning 540 million years of evolution (Ronshaugen et al., 2002) revealed that the strength of the activation domains changed during evolution, and that the location of the activation domain moved relative to conserved motifs and sub-domain organization (Liu et al., 2018). Similarly, fine-tuning of the interaction of the IDR of the kinase Pbs2 to specifically bind one SH3 domain among a context of many others (Zarrinpar et al., 2003; Kelil et al., 2016) was obtained through evolution. At the shorter timescale of the human life span, mutations accumulate, and while most are benign, stochastic changes can generate dysfunctional proteins, ultimately leading to disease. A proteome-wide study found that 22% of all human disease mutations locate to disordered regions (Uyar et al., 2014). Some disease-related mutations are SLiM-conserving, leaving affinity unperturbed, but with mutations located in the context resulting in altered specificity, cross-reactivity, and self-association as in the case of some neurodegenerative diseases (Uyar et al., 2014; Xiang et al., 2015). Finally, on one of the shorter time scales represented by the biological clock – the circadian rhythm – which is physiological processes happening on a 24-h cycle (Bell-Pedersen et al., 2005), the external context of IDPs differs due to changes in the available interactome. In mammals, the cycle is largely controlled by a heterodimer constituted of circadian locomotor output cycles kaput (CLOCK) and the TF brain and muscle arnt-like protein-1 (BMAL1) (Rey et al., 2011; Huang et al., 2012) and here, BMAL1 operates in different contexts depending on the time of day. CLOCK:BMAL1 regulates the transcription of thousands of different genes and the main regulators of CLOCK:BMAL1 activity are the proteins period 1 and 2 (Per1 and Per2) and cryptochrome 1 and 2 (Cry1 and Cry2), the genes of which are themselves regulated by CLOCK:BMAL1 (Gekakis et al., 1998). Per and Cry dimerize, interact with CLOCK:BMAL1 and inhibit transcriptional activity (Partch et al., 2014) in a negative feedback loop (Gekakis et al., 1998). Intriguingly, the activation domain of BMAL1 is further regulated by a proline switch, in which the Pro isomerizes between *cis*- and *trans*-conformations. Even though the Pro is the penultimate C-terminal residue, it still has a significant impact on the timekeeping ability of BMAL1. When the switch is locked in *trans*, the circadian rhythm is shortened to an extent comparable to deleting the switch, and when cyclophilins, a family of *cis*–*trans* peptidyl-prolyl isomerases are inhibited, the rhythm is prolonged (Figure 2) (Gustafson et al., 2017). This is suggesting that either the dynamics of the conformations is important or that the *cis* conformation is necessary for BMAL1 function. The fluctuation in CLOCK:BMAL1 activity that happens on a 24-h basis is dependent on changes in access to interaction partners, and in this way the context impacts the activity of the cell, contributing to the difference in organismal behavior during night and day.

In conclusion, the spatiotemporal context affects how disorder-based interactions operate, and with this in mind, we will continue with a more direct focus on the physicochemical context of the IDP itself.

## The Flanking Regions as Context

As defined above, flanking regions are here considered to be the ±20 residues N- and C-terminal to a SLiM or main contact site (Figure 1B), and may or may not contribute to binding affinity, specificity, and regulation of an interaction. Although rarely comprising 20 residues, the flanking regions may have strong modulatory impact on interactions, and they have been suggested to possess specific amino acid compositions (Fuxreiter et al., 2007). In this section, several examples of affinity modulation by flanking regions will be highlighted, as well as examples where the flanking regions play regulatory roles.

### Flanking Regions in Targeting

Many essential SLiMs have been identified in proteins destined for membranes. Acidic di-Leu- and Tyr-based internalization motifs are prominent examples of trafficking signals, which are involved in recruitment of clathrin-coated vesicles to membranes (Heilker et al., 1999). Their functional potency is illustrated by substitutions in their IDRs causing the syndrome glucose transporter causative of GLUT1 deficiency by creating di-Leu motifs (Meyer et al., 2018). Structural analysis of the AP2 clathrin adaptor complex with a bound di-Leu peptide revealed the functional importance of the residues of the core motif [DE]xxxL[LI] (Table 1) (Kelly et al., 2008). However, based on structural analysis, the ensemble context of the core motif, including the two residues C-terminal and a phosphorylated Ser five residues N-terminal of the first leucine were also suggested to affect binding (Kelly et al., 2008) (Table 1). Similarly, biochemical and structural analysis of the interactions between the di-Leu motif of the cation-dependent mannose 6-phosphate receptor and GGA proteins, implicated in protein trafficking between the Golgi apparatus and endosomes, revealed that both the N-and C-terminal di-Leu motif flanking regions influenced binding affinities. Thus, binding to the GGA proteins requires a precise spacing between the di-Leu and the free C-terminus (Misra et al., 2002) (Table 1).

**TABLE 1:** Selected examples of SLiMs affected by flanks and contexts to regulate function.

Core motif*	Parent protein	Extension of core motif by flanks and context	Function of motif	Function of flanks and context	References
PxxP	Pbs2	Various flanks	Binding of SH3 domains	Negative selection to increase specificity	Zarrinpar et al., 2003; Kelil et al., 2016
IxxLL	BMAL1	Very C-terminal residues, 20 positions from IxxLL	Binding of Cry and CBP to regulate the circadian rhythm	Allosteric regulation to regulate transcription in negative feed-back loop	Heery et al., 1997; Garg et al., 2019
[DE]xxxL[LI]	CD4	pS[DE]xxxL[LI]xx	Trafficking motif which binds clathrin adaptor proteins	Modulation of affinity	Kelly et al., 2008
[DE]xxLL	Mannose 6-phosphate receptor	xx[DE]xxLLxx-COOH	Trafficking between Golgi and endosomes	Modulation of affinity	Doray et al., 2002; Misra et al., 2002
[KR]DEL-COOH	Various ER-localized proteins	xx[KR]DEL-COOH/ ΩxΩKDEL KDEL-COOH	Recognition by KDEL receptors for ER retention	Modulation of specificity	Alanen et al., 2011; Mei et al., 2017
SxIP	CLIP-170	(Sx[IL]P)_*n*_CAP-G Sx[IL]PCAP-G	Targeting to microtubules	Multivalency to modulate affinity	Chen et al., 2019
QxxΦxx[FHT][FHY] QxxΦxx[FHT][FHY]-x_4_-[KR] Φ[KR]ΩΦΦ[KR]	PCNA partners (e.g., polymerases, E3-ligases, nucleases, helicases)	Charges (R/K) on each side	Replication fork localization motif to PCNA	Modulation of affinity by up to 4 orders of magnitude	Moldovan et al., 2007; Gilljam et al., 2009; Prestel et al., 2019
[IL]xCx[DE]	Host and viral interactors of Rb proteins	Negative charges	Binding to Rb family	Modulation of affinity	Palopoli et al., 2018
LxxIxE	Protein phosphatase 2A	Negative charges	PP2A binding motif	Affinity modulators	Hertz et al., 2016
EFFDAxE	OSBP	[ED]_6_EFFDAxE	Bridging between ER and other compartments	Initial low-affinity electrostatic binding	Loewen et al., 2003; Furuita et al., 2010
TQT	ASCIZ	Additional TQT	Binding of LC8 to regulate its level	Multivalency mediating positive and negative cooperativity	Clark et al., 2018
TPKK	p27^*Kip*1^	Charge distribution	Phosphorylation motif. Leads to degradation	Regulation of phophorylation	Das et al., 2016
GGxGxDx[Ω,Ψ],	Adenylate cyclase toxin	C-terminal disorder	Ca^2+^ binding and folding	Overall folding	Sotomayor Pérez et al., 2010
LP[Q/E]L	CITED2	α-helix-LP[Q/E]L	Binding to the TAZ1 domain of CBP	Anchoring and competition	Berlow et al., 2017
[DE]X[1,2][YF]X[1,4][DE]L	DREB2A and ANAC013	Conserved ID patterns	Binding to cellular hub RCD1	Negative and positive allostery	O’Shea et al., 2017; Christensen et al., 2019
RX_*n*1_R; *n*_1_ ≤ 2	Rpl5	RX_*n*1_Rx_*m*_RX_*n*1_R; *n*_1_ ≤ 2; *m* ≥ 2	Phase separation	Multivalency to modulate affinity	Mitrea et al., 2014, 2016
ΦΦWΦΦLF	GCN4	Additional hydrophobic patches	Transcriptional activation and phase separation	Multivalency needed for avidity in function	Warfield et al., 2014; Boija et al., 2018

*The order of the motifs in this table follows the order according to which they are mentioned in the main text.

Retrieval of many endoplasmic reticulum (ER)-resident proteins from post-ER compartments depends on a C-terminal [HKR]DEL motif (Majoul et al., 2001). Recent determination of the structure of the complex between the KDEL receptor and a KDEL peptide revealed how the SLiM residues contribute to binding (Bräuer et al., 2019). However, previous analysis of the [HKR]DEL flanks in human ER-localized proteins indicated that two residues N-terminal (positions −5 and −6) of the [HKR]DEL motif also played important roles for KDEL receptor recognition, and that different receptors have different preferences with respect to these positions (Alanen et al., 2011) (Table 1). Furthermore, simulation-guided studies revealed that aromatic residues in the extended motif ΩxΩKDEL also contributed to the interactions (Mei et al., 2017) (Table 1). Thus, here flanking regions may or may not be part of an extended motif, but constitute an important gearing of binding affinity as well as specificity (Figure 1B).

The microtubule network represents an essentially membrane-less compartment and is regulated by microtubule plus-end-tracking proteins (+TIPs) (Akhmanova and Yap, 2008; Jiang et al., 2012). Targeting of +TIPs to microtubules is mediated by SxIP anchoring motifs, found in, e.g., cytoplasmic linker protein 170 (CLIP-170), which engages in multivalent interactions with the protein End Binding 1 (EB1) (Chen et al., 2019). Whereas a central SxIP motif in CLIP-170 binds EB1 weakly, the so-called cytoskeleton-associated protein (CAP)-Gly domains are present in the flanking region and increase binding affinities by targeting different EB1 regions than the SxIP motif. Furthermore, additional N-terminal SxIP and SxIP-like motifs further increase binding affinities. In this binding model, the context of the central CLIP-170 SxIP motif provides avidity to the CLIP-170–EB1 interaction (Table 1 and Figures 1B,G) (Chen et al., 2019).

### Flanking Regions for Affinity Gearing

**FIGURE 3:**
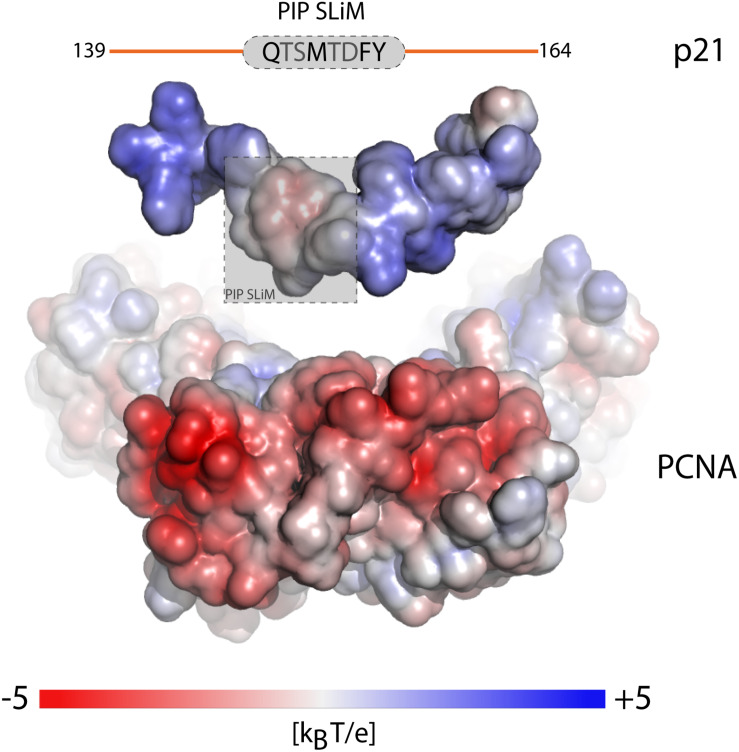
Context of the SLiM flanking regions fine tunes affinity. Electrostatic surface potential mapped onto the surface of the separated components of the p21-PCNA complex showing the outwards facing surface of one PCNA monomer of the trimer and the surface of a p21-peptide (PDB code 1AXC). Charge complementarity between positive charges in the flanking regions (orange) of the p21 PIP-degron motif (gray) and the highly negatively charged PCNA patches surrounding the binding pocket, modulates the binding affinity over four orders of magnitude. The figure was adapted from Figure 6C, originally published in Prestel et al. (2019), *Cell. Mol. Life Sci.*, 2019.

Proliferating cell nuclear antigen (PCNA) is an example where binding partners use a SLiM for anchoring (Moldovan et al., 2007). PCNA has an enormous interactome (Moldovan et al., 2007; Prestel et al., 2019) and common to its binding partners is the QxxΦxx[FHT][FHY] PIP-box motif of which variations are known, including the PIP-degron for degradation QTDΦxx[FHT][FHY]-X_4_-[KR] (Havens and Walter, 2009) (Table 1), and the APIM motif [KR]ΩΦ_2_[KR] for cytosolic partners (Gilljam et al., 2009; Sebesta et al., 2017). The motifs bind weakly in the low micromolar range, and the only currently known partner with nM affinity is p21 (Zheleva et al., 2000). Still, motifs harboring the canonical motif can have different affinities for PCNA, and the motif can degenerate, even to a degree where the similarity to the PIP-box is lost (Gilljam et al., 2009), but with retainment of similar affinities (Prestel et al., 2019). Recent work shed light on this paradox by showing that the flanking regions immediately surrounding the PIP-box had strong affinity-modulating activity through electrostatics. By increasing the number of positive charges in the flanking regions, the affinity for PCNA was modulated by up to four orders of magnitude (Figure 3 and Table 1) (Prestel et al., 2019). A similar effect was seen for pocket proteins of the retinoblastoma protein (Rb) family, that bind the SLiM LxCxE, present in host and viral interactors of the Rb family (Jones et al., 1990; Noval et al., 2013). Here, negative charges in the flanking regions act as affinity and specificity modulators (Figure 1B) (Palopoli et al., 2018), conversely regulated by introduction of negative charge from phosphorylation of binding regions on the Rbs (Knudsen and Wang, 1996). Similar charge modulation by flanks has been seen for the regulatory domain of protein phosphatase 2A (PP2A), where negative charges C-terminal to the SLiM (LxxIxE) enhanced affinity via electrostatics with a basic patch on PP2A; also mouldable by phosphorylation (Hertz et al., 2016) (Table 1), and for SH3 binding regions, where positive charges in the flanking regions modulate affinity (Figure 1B and Table 1) (Gorelik and Davidson, 2012; Teyra et al., 2012). In these cases, the context provides additional negative selection via mismatching flanking regions (Figure 1D and Table 1). How electrostatics contribute to binding, e.g., via salt bridge formation or mean-field type interactions (see below), is not clear.

### Flanking Regions as Motif Modulators

Communication between cell compartments also depend on context. Proteins with FFAT motifs (Loewen et al., 2003) communicate between the ER and other compartments by bridging (Murphy and Levine, 2016; Costello et al., 2017; Slee and Levine, 2019). The core of the FFAT motif, EFFDAxE, found in, e.g., oxysterol-binding protein (OSBP) (Furuita et al., 2010), is an extended region of seven residues (Table 1), the second and fifth of which bind into pockets in the integral ER membrane protein VAMP-associated protein (VAP) (Kaiser et al., 2005). Prior to binding of the core motif, an acidic tract positioned N-terminally to the motif mediates low-affinity electrostatic interactions with VAP (Figure 1B) (Furuita et al., 2010). Thus, here the context may lead to acceleration of the interaction via encounter complex formation.

The flanking regions may furthermore contribute mechanistically to disorder-based interactions. Cooperativity driven by flanking regions is elegantly demonstrated by the IDRs constituting the activation domain of the zinc finger TF ataxia telangiectasia mutated substrate Chk2-interacting Zn^2+^-finger protein (ASCIZ) (Clark et al., 2018). ASCIZ uses 11 out of 17 highly conserved TQT SLiMs to bind the dimeric hub protein LC8 (Ranaldi et al., 1994; Rapali et al., 2011), whereas Chia, another LC8 binding IDP uses three out of four TQT SLiMs to bind, whose affinities depend on the flanking regions in a non-predictable manner (Clark et al., 2016). The multivalency of ASCIZ and Chia allows both positive and negative cooperativity in its interaction with LC8. ASCIZ binding to LC8 generates a scaffold (Clark et al., 2015) onto which additional LC8s bind with increased affinity (Figure 1H and Table 1). Then, negative cooperativity regulates the formation of higher-order LC8 assemblies to ensure that low-occupancy complexes dominate at saturating concentrations of LC8 to prevent switching off transcription completely (Clark et al., 2018). Thus, for the disordered ASCIZ and Chia, both flanks and context affect binding affinity and *in vivo* regulation of activity.

Finally, when SLiMs are placed in pre-structured contexts, defined as molecular recognition features (MoRFs) (Malhis and Gsponer, 2015; Sharma et al., 2018) or pre-structured motifs (PreSMos) (Lee et al., 2012), the flanking regions can modulate the structural context of the motif and hence interactions. This has been exemplified by prolines in flanking regions impacting helicity, and it has by computation, e.g., been shown that when a proline was mutated in an N-terminal flanking region, helicity was significantly decreased, whereas when mutated in the C-terminal flanking region, the helical content increased (Lee et al., 2014). Proline structural modulation was later shown to impact target binding, with the P27A-variant of p53 binding more tightly to Mdm2 when helicity was boosted (Borcherds et al., 2014).

The examples given above are part of an emerging picture of the importance of the flanking context for SLiM-driven IDP interactions. They have in common that the aromatic and charged residues of the core SLiM motif are flanked by additional charged or aromatic residues that initiate and enhance binding as well as provide a platform for negative selections and cooperativity (Figures 1B,D,H). In this way, flanking regions – or interaction sites along the chain - may constitute an affinity gearing of the core SLiM.

## The Context of the Disordered Chain

Most SLiMs are embedded in disordered chains of various lengths, some as long as 500 residues (Christensen et al., 2019) and some as short as 30. Furthermore, some SLiMs and their disordered chain are connected to globular domains of different sizes and function, while others exist in a fully disordered setting. The properties of the chain (ordered, disordered, long, short, sequence-, and ensemble properties) are part of the context and may influence interactions. In the following we will highlight examples that illustrate how chain properties may modulate disorder-based interactions.

### The Context of Sequence Properties of the Chain

#### Influence of Amino Acid Composition on Chain Properties

Sequence properties are an important factor for compaction of disordered chains (Das et al., 2015; Martin et al., 2016, 2020). To date, various studies have assessed the sequence-encoded conformational preferences of IDPs, using concepts from polymer physics reviewed in Mao et al. (2013) and by combining several biophysical techniques with molecular simulations. These altogether point toward compositional parameters such as the net charge (Marsh and Forman-kay, 2010; Muller-Spath et al., 2010) or more specifically the distribution of charges (Das and Pappu, 2013), and proline residues (Marsh and Forman-kay, 2010; Martin et al., 2016) as being key determinants for their chain dimensions. Alterations to the net charge of IDPs, through for example alternative splicing or posttranslational modifications, can greatly affect their compaction and hence functions. A notable example was reported for the cyclin-dependent kinase inhibitor p27^*Kip*1^. Here, modifications of the conserved distribution of charged residues in the flanking region of the SLiM TPKK provided a mechanism for controlling the phosphorylation efficiency of the SLiM Thr, leading to degradation of p27^*Kip*1^, and thus being responsible for entry into the S-phase of the cell cycle (Das et al., 2016) (Table 1). As most IDPs are polyampholytes (Das et al., 2015), modifications to SLiM flanking regions modulating their charge state (by e.g., phosphorylation) may induce transitions in the conformational ensemble of the disordered chain enabling allosteric regulation of motif accessibility (Figure 1F). Interestingly, while hydrophobicity has been suggested to play a marginal role in IDR compaction (Marsh and Forman-kay, 2010), a correlation between the fraction and patterning of aromatic residues and chain compaction was recently established (Sørensen and Kjaergaard, 2019; Martin et al., 2020). IDPs can be highly phosphorylated (Iakoucheva et al., 2004), which further introduces an increased pH sensitivity of disorder-based interactions, as phosphate groups titrate in the physiological pH range (pKa pTyr = 5.83, pSer = 6.01, pThr = 6.30) (Hendus-Altenburger et al., 2019). Differential effects of IDP-phosphorylation have been observed, which may increase or decrease transient helicity in the vicinity of binding motifs, which in turn contributes to selection and deselection of binding partners as well as initiating degradation (Bah and Forman-Kay, 2016; Mylona et al., 2016; Hendus-Altenburger et al., 2017). Phosphorylation may also lead to global folding, as observed for the disordered eukaryotic translation initiation factor 4E binding protein 2 (4EBP2), which binds eukaryotic translation initiation factor 4E (eIF4E) and suppresses cap-dependent translation initiation. 4EBP2 folds upon phosphorylation of specific sites, and the more folded 4EBP2 becomes, the lower its affinity for eIF4E (Bah et al., 2015).

#### Influence of Amino Acid Composition and the Physicochemical Environment on Compaction

Due to their higher degree of solvent-contact, IDPs are expected to be more sensitive to changes in the environment and consequently, so will their interactions. Temperature, pH, ions, solvent, salt concentration, and viscosity influence chain compaction as well as IDP interactions. For example, pH is highly connected to cellular location, as, e.g., intracellular pHs range from pH 4.7 in the lysosomes to pH 8.0 in the mitochondria. Thus, depending on location (Casey et al., 2010), IDPs may compact, gain secondary structure, or engage in folding or partial folding when the pH changes. Prothymosin-α (ProTα), a highly acidic, nuclear IDP, is expanded at neutral pH, but compacts as the pH decreases (Uversky et al., 1999). Similarly, proteins with high overall positive charge, such as core histones, partially fold when pH increases, affecting DNA binding (Hansen et al., 1998; Munishkina et al., 2004). Another example is the highly positively charged myelin basic protein (MBP), essential for the formation and stability of the myelin sheath in the central nervous system (Majava et al., 2008; De Avila et al., 2014). MBP is disordered, but folds into an α-helical structure upon electrostatic interaction with the membrane (Polverini et al., 1999; Harauz et al., 2009), a transition highly regulated by salt and Ca^2+^ (Raasakka et al., 2019). A similar behavior was also observed for the IDP α-synuclein (aSN) (Georgieva et al., 2008; Fusco et al., 2014; Cholak et al., 2020).

Chain compaction may further be affected by metal ions, as observed for Zn^2+^ binding to ProTα, and to histatin, a small peptide of the mouth, here promoting formation of higher order structures (Cragnell et al., 2019). For both IDPs, these are Zn^2+^-specific effects (Yi et al., 2007; Cragnell et al., 2019). Similarly, Ca^2+^-binding to the C-terminal of aSN is specific, with other divalent cations showing much lower affinity (Lowe et al., 2004). For the disordered region of the adenylate cyclase toxin (Chenal et al., 2009), Ca^2+^ binding to >40 repeats in toxin motifs (RTX) GGxGxDx[Ω,Ψ] led to folding into a β-roll structure (Sotomayor Pérez et al., 2010) central to its secretion (O’Brien et al., 2018) (Table 1). In this case, binding to the RTX motifs was not enough to induce folding, but required the presence of a C-terminal disordered flanking region (Sotomayor Pérez et al., 2010). However, ions not only affect structure and conformational properties via distinct binding, but their presence can also lead to changes in the ensemble, as shown for five different IDPs, which all expanded due to an increase in salt concentration (Vancraenenbroeck et al., 2019). Salts also tune disorder-based affinities and binding kinetics, as demonstrated for two different IDPs (Wicky et al., 2017), where the observed effects were dependent on the specific ions and not simply correlated to the ionic strength.

Thus, the conformational space sampled by the disordered chain is intimately linked to its physicochemical properties and to those of the surrounding environment. This ensemble can be shifted by alterations to the chain properties by extrinsic factors such as temperature, ions and salt concentration or by intrinsic factors such as post translational modifications or changes in protonation state.

### The Context of Chain Dynamics

#### Fast Chain Dynamics in Specialized Interaction Mechanisms

Recently, it has become clear that high degrees of fast, long-range dynamics may be retained in IDP complexes, and for some of the more recently discovered IDP interaction mechanisms, the context of the chain dynamics is an important prerequisite for interaction. This is the case for the interaction of the intrinsically disordered Phe-Gly-rich nucleoporins (FG-Nups) and nuclear transport receptors (NTRs). FG-Nups fill the central cavity of the nuclear pore complex, allowing passage of large molecules only when bound to NTRs. The interaction of FG-Nups with NTRs occur through the FG-SLiMs, which individually have NTR-affinities in the millimolar range (Milles et al., 2015). However, by combining many FG-SLiMs, an affinity in the nM-range is achieved (Hough et al., 2015). This avidity effect (Figure 1G) is facilitated by the retained flexibility and plasticity in the bound state of the FG-Nups, resulting in fast binding and unbinding of the individual motifs to different sites. The resulting highly dynamic multivalent interaction type enables high specificity along with a fast transport rate though the pore (Milles et al., 2015). Another example is the pM-affinity electrostatically driven interaction between H1 and its chaperone ProTα (Borgia et al., 2018). Here the retainment of flexibility of the two IDPs as well as their long-range dynamics in the complex allow rapid interconversion between many different conformations on the 100-ns time scale, facilitating a mean-field type electrostatic interaction between all charges (Borgia et al., 2018). Finally, pre-existence of chain disorder may allow for special cases of allovalency, as shown for the IDP Sic1 binding to cell-division control protein 4 (Cdc4). Here, multivalency from several identical sites distributed along a disordered chain binding to the same binding site increased binding affinity *via* allovalency (Figure 1I). The binding is cooperative, with almost no detectable binding until a sixth arbitrary site among ten becomes phosphorylated, producing strong binding (Mittag et al., 2008); a scenario only possible in the context of a flexible, dynamic chain (Locasale, 2008).

#### Chain Dynamics in Partner Selection

**FIGURE 4:**
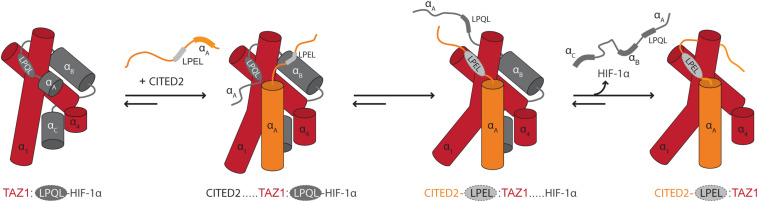
Modulation of binding by chain dynamics. The flexible nature of HIF-1α bound to TAZ1 allows the transactivation domain of CITED2 to gain access to TAZ1 through its N-terminal region. This results in the formation of a ternary complex which induces an allosteric conformational change in TAZ1, disfavoring/out-competing HIF-1α binding. The CITED2 LPEL-SLiM and flanking α_*A*_ helical region act cooperatively to displace HIF-1α from the shared binding site for the LP(Q/E)L SLiM and the restricted motions adopted in the bound state minimize competition for binding. In this schematic illustration, TAZ1 is depicted in red, HIF-1α is dark gray and the bound LP(Q/E)L SLiM is light gray with a dashed outline. The SLiM-flanking regions in CITED2 are shown in orange. Adapted from Figure 4, originally published in Berlow et al. (2017).

The context of chain dynamics also partakes in the recognition and competitive interactions of disordered TFs and hub proteins, as exemplified by the interaction of the TFs hypoxia-inducible factor (HIF)-1α and CBP/P300 interacting *trans*-activator with ED-rich tail domain 2 (CITED2) with the telomere length regulator 1 (TAZ1) domain of the general transcriptional co-regulatory CBP (Figure 1E) (Berlow et al., 2017). The two TFs use the core motif LP[Q/E]L for binding to the same site on TAZ1 (Table 1), and bind with close to identical affinities (De Guzman et al., 2004; Berlow et al., 2017). Still, CITED2 out-competes HIF-1α for binding to TAZ1 (Berlow et al., 2017). The mechanism of this displacement has mainly been explained from detailed NMR studies (Berlow et al., 2017, 2019), according to which differences in the dynamic profiles on the pico- to nanosecond time scale of the bound TFs modulate competition for TAZ1. Both TFs fold upon binding to TAZ1, but HIF-1α retains a high degree of flexibility in complex with TAZ1, while CITED2 uniformly rigidifies (Berlow et al., 2019). The dynamics particularly of the N-terminal region of bound HIF-1α was suggested to allow CITED2 to access a key surface on TAZ1, promoting ternary complex formation and eventually displacement of HIF-1α. Simultaneously, the rigidification of key regions of CITED2 upon binding prevents HIF-1α from back-competing (Figure 4). When characterizing TAZ1 in the free and bound states, the structure of TAZ1 was nearly identical in the HIF-1α- and CITED2 bound states, but TAZ1 became more rigid in complex with CITED2. The rigidification occurred particularly in the binding region and regions undergoing conformational changes between HIF-1α- and CITED2-bound states, suggesting tuning of TAZ1 backbone dynamics to discriminate between disordered partners (Berlow et al., 2019). Thus, backbone dynamics in folded hubs as well as their disordered partners play roles in partner selection and complex stability.

In combination, these studies have highlighted that the context of the inherent flexibility and dynamic properties of IDPs may allow for previously unknown binding- and competition mechanisms as well as bridge between the fast binding and high affinity needed in regulatory networks.

### Ensemble Redistribution and Allostery by Context

#### Correlated Fluctuations in Conformational Ensembles

Emerging evidence suggests that the context of the ensemble of the entire IDP may be of key importance to understanding disorder-based interactions. Recently, new advanced applications of techniques such as fast field cycling (FFC) relaxometry (Parigi et al., 2014) and paramagnetic relaxation enhancement (PRE) NMR spectroscopy (Kurzbach et al., 2016) have demonstrated the existence of correlated dynamics in IDPs over long sequential distances. Using FFC relaxometry, it was shown for four IDPs that slow reorientations on the several nanosecond time scale occurred in the chain, without specific residue interactions, but rather based on friction-mediated coupling (Parigi et al., 2014). Providing further details, a method based on PRE NMR, termed paramagnetic relaxation interference (PRI), allowed detection of correlated motions through covariance analysis of the effect of adding two paramagnetic labels to the same chain (Kurzbach et al., 2016, 2017). Utilizing this technique, Beier et al. (2018) showed that aSN and osteopontin display “energetic frustration” in their free states, which is the inability to fulfill conflicting energy requirements at the same time. Both proteins exhibited both correlated, anti-correlated and uncorrelated long-range chain fluctuations (Beier et al., 2018), with two residues being correlated if they display concerted motions or undergo simultaneous transitions between different conformational states, anti-correlated if they have anti-correlated fluctuations or structural changes due to, e.g., mutually exclusive conformational sub-states, and uncorrelated if they are independent. Interestingly, the correlation of motions changed for both IDPs upon partner binding (Beier et al., 2018). When the PRI analysis was performed on aSN bound to calmodulin and osteopontin bound to heparin, the anti-correlated fluctuations, or energetic couplings, of their free states were relieved. For aSN, this meant that sub-states of the aggregation-prone region were relieved of negative coupling, which the authors suggested may explain how calmodulin facilitates aggregation of aSN (Martinez et al., 2003).

#### Regulation by Dynamic Allostery

Proteins with energetically frustrated IDRs can mediate allosteric regulation (Garcia-Pino et al., 2010; Ferreon et al., 2013), which play a central role in orchestrating cellular signaling networks (Nussinov et al., 2013). Theoretical models for proteins where at least one of the coupled sites is an IDR, assume that allosteric modulation is mediated via many conformers, where fluctuations among the conformers of one site can modulate the functional output of another site through energetic coupling (Hilser and Thompson, 2007) (Figure 1F). According to this allosteric ensemble model, the flexibility of disorder allows complex allosteric behavior of IDRs in fine-tuning regulatory interactions (Hilser and Thompson, 2007; Motlagh et al., 2014). The importance of such energetic frustration in IDP interactions has also been demonstrated from work on the glucocorticoid receptor (GR), where the disordered domain allosterically controls function by principles of energetic frustration (Li et al., 2018). GR has three domains: the C-terminal, folded DNA-binding domain (DBD), the disordered F-domain, and the N-terminal disordered R-domain. Binding of DNA to the DBD results in positive coupling to the F-domain ensemble, which is shifted toward stabilization of its folded form, in turn increasing its transcriptional activity. However, the DNA-bound DBD is also negatively coupled to the R-domain, shifting its ensemble to a state that destabilizes the F-domain. The net effect of DNA binding to the DBD on transcriptional activity of the full-length GR is hence a balance between the strength of the two couplings (Li et al., 2018). Here, allostery is not an effect of a mechanical pathway between two sites, but rather by the energetic balance within the conformational ensemble, represented by changes in the population of states (Hilser and Thompson, 2007). Through this ensemble-mediated mechanism, multidomain proteins are suggested to exist in an ensemble of states poised to respond to binding. Binding leads to an ensemble redistribution, with a corresponding change of ensemble properties of the intact protein (Hilser and Thompson, 2007). For GR, signaling is tuned by changing the length of the intrinsically disordered context through translational isoforms, resulting in modulation of the degree of energetic frustration (Li et al., 2012, 2018).

Examples of flanking regions and chain contexts impacting binding by mechanisms involving dynamic allosteric regulation are accumulating. The competitive interactions of the TFs HIF-1α and CITED2 with the TAZ1 domain described in the previous section are for example an important demonstration of allosteric effects of SLiM flanking regions (Berlow et al., 2017, 2019) (Figure 1G). This example underscores how the dynamics and structure of the flanking regions of a SLiM may be even more important than the SLiM itself. For the plant TFs dehydration response element binding proteins 2A (DREB2A) and ANAC013, which both use the [DE]X[1,2][YF]X[1,4][DE] SLiM for binding to the cellular hub protein Radical Induced Cell Death1 (Bugge et al., 2018), disordered regions surrounding the binding motif exert positive and negative allosteric effects on binding, respectively (O’Shea et al., 2017) (Figure 1G and Table 1), possibly reflected in the function-related conservation of the disorder-order profiles for the NAC transcription factors (Stender et al., 2015). These effects could also be explained by the ensemble allosteric model as derived for GR. Thus, these examples show how flanking regions and chain contexts can contain sub-regions that are coupled to the SLiM, enabling allosteric modulation of the stability and accessibility of this site as well as adding avidity effects (Figures 1E–G).

### The Context of Phase Separation

The formation of self-assembled, membrane-less organelles through liquid-liquid phase separation (LLPS) of proteins creates special contexts for IDP-based interactions and partitions with specific functions [for reviews, see Mitrea and Kriwacki (2016), Zaslavsky et al. (2018), Alberti et al. (2019)]. A SLiM may itself be necessary and responsible for leading IDPs into LLPS (Figure 1J), but conversely, LLPS may obstruct any other SLiM from engaging in interactions. The nucleolus, a membrane-less compartment, is the site of ribosome biogenesis (Boisvert et al., 2007). Here, nucleophosmin (NPM1) is present at high concentrations, and, using its N-terminal domain, interacts with multiple other proteins via their Arg-rich SLiMs (R-motifs) (Figure 1J and Table 1) (Mitrea et al., 2014). Localization of NPM1 within nucleoli depends upon its ability to undergo LLPS with Arg-motif containing proteins and ribosomal RNA (Mitrea et al., 2016; Banani et al., 2017). Whereas a peptide derived from the ribosomal protein L5 (RPL5) with a single R-motif was sufficient for detectable binding to NPM1, at least two Arg-motifs were needed for LLPS, demonstrating the need of Arg-motif multivalency (Figure 1H) (Mitrea et al., 2016). Thus, the chain context contributes by increasing the number of motifs to establish different features that are not extractable from a single motif on its own.

Additional examples illustrate how IDRs with multiple interacting motifs can participate in LLPS mediated by weak multivalent interactions (Boija et al., 2018; Alberti et al., 2019). This is the case for, e.g., the activation domains of gene-specific TFs such as the yeast TF GCN4 (Boija et al., 2018). The activation activity of GCN4 depends on the SLiM ΦΦWΦΦLF (Table 1) (Warfield et al., 2014; Staby et al., 2017). However, this motif is part of a region with several hydrophobic patches, and GCN4 binds the Mediator co-activator component MED15 via multiple, low-affinity interactions, which additively contribute to activation activity (Drysdale et al., 1995; Warfield et al., 2014; Staller et al., 2018). Furthermore, for a mutant of GCN4 in which the aromatic residues of the hydrophobic patches were changed to Ala, incorporation into MED15 droplets was attenuated (Boija et al., 2018). Jointly, the results suggest that GCN4 and other TFs activate genes through the phase separating capacity of motif-centered, context regulated interactions by their activation domains (Boija et al., 2018).

In phase separation, the context of the disordered chain also has implications for interactions as explained by the stickers-and-spacers model of phase separation developed based on studies of RNA-binding proteins with prion-like domains (Wang et al., 2018; Martin et al., 2020). According to this model, the number (valence) of aromatic residues (stickers) and the patterning of the stickers, governed by the spacers, determine the phase behavior of prion-like domains to the extent that a numerical stickers-and-spacers model enables prediction of binodals/phase behavior from amino acid sequence (Martin et al., 2020). Future studies will have to show to what extent the stickers can be SLiMs, and not only individual residues, as well as reveal the characteristics of the spacers (Harmon et al., 2017).

**Box 1 | Outstanding questions.**

• How do we differentiate and define SLiMs, flanking regions and chain context?
• How do we study and analyse the effects of flanking regions and chain context systematically?
• Are flanking regions and chain context evolutionary hot spots for hub interactions?
• Are flanking regions and chain context in some complexes more important in regulation than the SLiM itself?
• May flanking regions and chain context partake in new types of interaction mechanisms?
• Is chain dynamics an important contributor in interaction network fidelity?
• To which extent may IDP interactions be understood out of the full chain context?

## Conclusion and Outstanding Questions

With the discovery of IDPs, the palette of interaction mechanisms is continuously expanding and forcing us to rethink protein interactions. Already in the 1970s, the importance of context in the understanding of proteins was formulated by Christian B. Anfinsen stating “*that the native conformation is determined by the totality of interatomic interactions and hence by the amino acid sequence, in a given environment*” (Anfinsen et al., 1961; Anfinsen, 1973). Thus, context has long been considered as part of the equation. For IDPs, the role of the environment, which is also its own disordered chain, may amplify due to their different properties compared to globular proteins. With a mouldable chain, context becomes broader than the chain itself, providing a much stronger contribution to regulation of disorder-based interactions. However, with the few examples highlighted in this paper, we need many more to be able to fully comprehend the role of the context in the orchestration of interactions involving IDPs. Hopefully, the present review has made it clear that the binding of many disordered proteins depends heavily on the context. Binding of many – if not all – SLiMs involves contributions from the flanking regions and/or the context. These contributions may be electrostatic in nature acting through dense regions of similar charges, either highly negatively charged, as for the flanks of the SLiM LxCxE of the Rb binding proteins (Palopoli et al., 2018), or highly positively charged as for the PCNA binding PIP-box (Prestel et al., 2019). However, the flanking regions may also have a highly hydrophobic character, as for the flanks of the [HKR]DEL SLiM (Alanen et al., 2011). Finally, the structure and dynamics of the flanks may be adding to competitions, cooperativity and allosteric regulation (Berlow et al., 2017; Li et al., 2017) as well as to ensure proper orientation and increase the speed of interaction, as discussed previously (Fuxreiter et al., 2007). In these cases, the role of the context and the flanking regions have been shown to modulate the affinity and have regulatory potential (Table 1). Indeed, as outlined above, there seems to be a surprising dependence of affinity and complex stability on the flanking regions. The thermodynamic details and structural requirements of flanking region interactions are largely unknown and represent an exciting challenge for the biophysical community.

Several questions remain outstanding (Box 1). The questions mainly address how interactions beyond the central contact points contribute to disorder-based interactions. Notably, the properties and importance of the flanking regions and of the disordered context have not been systematically addressed. Is it possible that our view on SLiMs is too restricted and that flanking regions or chain context should be considered as a true part of the motif and that they play roles in addition to modulation? Similarly, is it possible that context plays hitherto unrecognized roles as in, e.g., forming interactions in the unbound state to limit accessibility allosterically or participating in unrecognized mechanisms? Finally, have flanking regions and chain contexts developed to be meaningful for binding in those cases where a motif is overlapping/combined from several competing motifs? Once canonicity of a motif is lost, the flanking regions and the chain context could become evolutionary hot spots for maintaining binding to both – or more – partners. The examples in the present review testifies that work is being done to probe the effect of the context, but the journey has just begun. So, although we here highlighted the importance of flanking regions and the disordered chain properties in mediating regulatory function to IDP-based interactions, a huge knowledge void exists as to how these quantitatively and mechanistically contribute to binding, and more systematic studies as well as studies *in vivo* are highly warranted.

## Author Contributions

KB, IB, CF, JD, JL, JO, KS, and BK contributed to the conceptual developments. JL, JD, CF, and IB contributed with first drafts of the paragraphs describing the external context and sequence properties, and the manuscript was written by KB, JO, KS, and BK with input from and discussion with all authors.

## Conflict of Interest

The authors declare that the research was conducted in the absence of any commercial or financial relationships that could be construed as a potential conflict of interest.
